# Examination of the hygienic status of selected organic enrichment materials used in pig farming with special emphasis on pathogenic bacteria

**DOI:** 10.1186/s40813-018-0100-y

**Published:** 2018-10-11

**Authors:** Krista Marie Wagner, Jochen Schulz, Nicole Kemper

**Affiliations:** Institute for Animal Hygiene, Animal Welfare and Farm Animal Behaviour (ITTN), University of Veterinary Medicine Hannover, Foundation, Bischofsholer Damm 15 (Building 116), 30173 Hannover, Germany

**Keywords:** Hygiene, Mycobacteria, Pig husbandry, Peat, Straw

## Abstract

**Background:**

Enrichment materials for pigs, particularly organic materials, are becoming increasingly important in order to reduce abnormal behaviour such as tail biting. However, potential health risks posed by these materials (such as the introduction of pathogens into the herd) have not been sufficiently studied to date. Therefore, 21 different organic materials used as enrichment materials in pig farming were tested for total viable count of mesophilic bacteria, moulds, coliforms, *Escherichia coli*, *Klebsiella* spp., *Yersinia* spp., *Salmonella* spp., methicillin-resistant *Staphylococcus aureus*, and *Mycobacterium* spp. Additionally, dry matter content and water activity were determined.

**Results:**

The materials differed considerably in their hygienic status. In three materials, no microorganisms were detected. However, the bacterial count in the other materials ranged up to 7.89 log_10_ cfu/g dry matter (maize silage). The highest coliform and mould counts were found in hay (6.45 and 6.94 log_10_ cfu/g dry matter, respectively). Important bacteria presenting a risk to human or animal health such as *Escherichia coli*, *Klebsiella* spp., *Yersinia* spp., *Salmonella* spp., and methicillin-resistant *Staphylococcus aureus* were not detected in any of the materials. Hemp straw contained *Mycobacterium smegmatis,* and peat was contaminated with *Mycobacterium avium* and *Mycobacterium vulneris*.

**Conclusions:**

Most of the tested organic materials are probably not likely to pose a hygienic risk to pigs and are suitable as enrichment material. Nonetheless the detected mycobacteria rule out peat as being a safe and hygienic enrichment material.

## Background

Exploratory behaviour is of great importance to pigs. This occupies most of their daytime activity if they are kept in a semi-natural environment [1]. In barren environments such as intensive pig holdings, in which the need to perform exploratory behaviour is not fulfilled, it was found that pigs redirect this behaviour towards pen equipment and pen mates [2]. In the same publication, multiple studies were reviewed showing a reduction in this redirected behaviour (such as tail biting) that was caused by providing enrichment material [2]. In addition, farmers in the European Union (EU) are legally obliged to permanently provide their pigs with manipulable material that does not compromise the animals’ health [3]. The exemplarily mentioned materials in Council Directive 2008/120/EC [3] are without exception organic materials; namely straw, hay, wood, sawdust, mushroom compost, and peat. According to Studnitz et al. [2], suitable enrichment material should be complex, changeable, destructible and contain edible parts, all of which solely applies to organic materials. Similar requirements were recently stated in the Commission Recommendation 2016/336/EU [4] that optimal materials should be edible, chewable, investigable, and manipulable. However, the EFSA [5] warned about adverse effects of using enrichment material. Contamination of the materials with infectious agents such as *Yersinia enterocolitica*, Oesophagostomum or mycobacteria, mycotoxins, or chemical compounds such as chloramphenicol is possible [5]. Additionally, destructible materials might induce problems with the manure system, thus decreasing hygiene and air quality in the stable [5]. The aim of the present study was to investigate the hygienic status and contamination with potentially pathogenic microorganisms of diverse organic enrichment materials. Since scientific literature in addition to legislation evaluate organic material as being able to satisfy the behavioural needs of pigs, this study concentrated on examining organic materials with respect to the way in which they influenced pigs’ behaviour. The hypothesis of the study was that certain organic enrichment materials are a source of pathogenic bacteria that could cause infection in pigs. Based on this, suitable enrichment materials from a hygienic point of view should be recommended.

## Results

The dry matter (DM) content of most materials was between 85.61% (hay) and 98.58% (millings) and a water activity ranging from 0.36 (millings) to 0.70 (wood granulate) (Table 1).

**Table 1:** Water activity and dry matter content of the tested organic materials

Material	Water activity (a_w_)	Dry matter content (%)
Wooden materials		
Wood granulate	0.70	85.80
Wood shavings	0.60	86.81
Sawdust	0.55	89.79
Millings	0.36	98.58
Loose straw and hay		
Flax straw	0.65	85.70
Wheat, rye, triticale straw meal	0.52	87.91
Alfalfa hay	0.53	88.45
Rye straw meal	0.48	88.29
Hemp straw	0.61	86.85
Hay (from farm)	0.59	85.61
Wheat straw (from farm)	0.61	86.78
Compressed straw and hay		
Compressed straw cylinder	0.42	89.17
Straw pellets	0.53	90.26
Hay pellets	0.41	90.92
Miscanthus cylinder	0.51	91.00
Miscellaneous		
Beet pulp with molasses	0.50	93.76
Maize pellets	0.54	93.56
Peat	0.95	31.83
Lick block	n.a. ^a^	n.a. ^a^
Lignocellulose	0.59	88.48
Maize silage	0.94	27.13

^a^n.a., not applicable

However, peat and maize silage showed a considerably higher water activity (0.95 and 0.94, respectively), and had low DM contents of 31.83% and 27.13%, respectively. *Escherichia coli*, *Klebsiella* species (spp.), *Yersinia* spp., *Salmonella* spp., and methicillin-resistant *Staphylococcus aureus* (MRSA) were not detected in any of the tested materials. *Mycobacteria* (*M*.) were found in two of the tested materials. The hemp straw contained *M*. *smegmatis*, and the peat was contaminated with *M*. *avium* and *M*. *vulneris* (Table 2).

**Table 2:** Bacterial occurrence in the tested organic materials

Analysed bacteria	Organic material
*Escherichia coli*	not detected
*Klebsiella* spp.	not detected
*Yersinia* spp.	not detected
*Salmonella* spp.	not detected
methicillin-resistant *Staphylococcus aureus*	not detected
Mycobacteria	
*Mycobacterium smegmatis*	Hemp straw
*Mycobacterium avium*	Peat
*Mycobacterium vulneris*	Peat

The results for total viable counts (TVC) and coliform and mould counts differed considerably as shown in Table 3. In the wood shavings, beet pulp, and lignocellulose litter, no microorganisms were detected at all. The highest TVC was found in the maize silage (7.89 log_10_ colony forming units [cfu]/g DM), while the hay exceeded the other materials with 6.45 log_10_ cfu coliforms/g DM. One third of the materials did not contain moulds; the maximum level of 6.94 log_10_ cfu/g DM was also found in hay.

**Table 3:** Overview of the tested organic materials from different sources (1 = commercially available, 2 = farm-based) and microbial counts (log_10_ cfu/g DM) as total viable count (TVC) and coliform and mould counts; LOD = limit of detection

Material	Source*	TVC^a, b^	Coliforms^b^	Moulds^b^
Wood-based materials				
Wood granulate	1	< 3.70	< LOD	< 3.70
Wood shavings	1	< LOD	< LOD	< LOD
Sawdust	1	< 3.73	< LOD	< 3.73
Millings	1	3.81	< 3.69	< 3.69
Loose straw and hay				
Flax (*Linum* L.) straw	1	7.04	5.78	4.73
Wheat, (*Triticum* L.), rye (*Secale cereal* L.), triticale (*Triticale* Tscherm.-Seys. ex Müntzing) meal	1	6.99	6.20	4.65
Alfalfa *Medicago sativa* L.) hay mixed with green harvested oats (*Avena* L.) and clover (*Trifolium* L.) and treated with molasses and vegetable oil	1	5.66	3.86	< 3.73
Rye (*Secale cereal* L.) straw meal	1	6.81	6.23	5.34
Hemp (*Cannabis* L.) straw	1	6.76	5.43	4.38
Grass hay	2	7.54	6.45	6.94
Wheat (*Triticum* L.) straw	2	7.63	5.96	5.94
Compressed straw and hay				
Compressed wheat (*Triticum* L.) and rape (*Brassica napus* L.) cylinder	1	4.34	< LOD	< LOD
Wheat (*Triticum* L.) pellets	1	5.43	< LOD	< LOD
Grass and herb hay pellets	1	7.00	< LOD	< LOD
Miscanthus (*Miscanthus* ANDERSSON) cylinder	1	5.04	< 3.72	4.15
Miscellaneous				
Sugar beet (*Beta vulgaris* subsp. *vulgaris* L.) pulp with molasses	1	< LOD	< LOD	< LOD
Maize (*Zea mays* L.) pellets	1	4.11	< LOD	< LOD
Peat (rooting material for piglets)	1	6.65	< LOD	4.91
Lick block	1	3.70	< LOD	< LOD
Lignocellulose made of dehydrated molasses, vegetable fat and mineral nutrients	1	< LOD	< LOD	< LOD
Maize (*Zea mays* L.) silage	2	7.89	< LOD	< 4.15

## Discussion

The tested enrichment materials differed considerably in their material type and source and also in their hygienic status. They were chosen due to their practical relevance and their commercial availability. With regard to practical relevance, hay and straw harvested on farms are widely used, but differ greatly in their quality due to weather, harvest, storage conditions, and other factors and are therefore not comparable. Thus, in this study hay, straw, and also maize silage were obtained from one example farm only. Those results are only intended to be a preliminary guide of what values can be expected and cannot be extrapolated to materials from other farms or material harvested at other points in time. However, in the commercially available materials with different standard processing steps that are done before they are sold, comparability would be desirable. In this context, one limitation of this study was the purchase of most of the materials from only one commercial source. For materials from other sources, results might differ, but as the manufacturing process is of high importance, this study provides the first results for materials produced under similar conditions, irrespective of the country of production. During production, more precisely during the pelleting process, the material is exposed to heat, pressure, shear forces, and water. Numerous studies have shown that pelleting reduces the contamination with vegetative microbes mainly due to a reduction in water activity [6]. Water activity characterizes the availability of free water in the material, which is essential for bacterial survival and growth.

Referring to the results in detail, due to the study design, only descriptive statistical analyses were performed. The study was intended as a pilot study to provide the first insights into this relevant topic. Regarding coliforms in general, the level of contamination in enrichment materials made of wood was below the median value of 281 wood shavings with 7.9 × 10^5^ cfu/g and 1.2 × 10^3^ cfu/g for TVC and coliform count, respectively [7]. It has been shown that wood, depending on several factors such as wood species and ambient temperature, can reduce the survival of bacterial species such as *Escherichia coli* [8] via hygroscopic effects and wood extractives. Furthermore, adherence of microorganisms to the surface of wood is discussed as a reason for reduced recovery. However, Vainio-Kaila et al. [9] demonstrated that this adherence can be avoided by vortexing the samples. Thus, adherence should not have a major influence on the results of this study. Kristula et al. [10] considered coliform counts in bedding material exceeding 10^6^ cfu/g as a potential cause of mastitis in dairy cows. Even though three of the loose straw and hay materials in this study had a higher coliform burden than the threshold of 10^6^ cfu/g described by Kristula et al. [10], it is questionable whether this threshold is applicable in pig husbandry. The total viable count in the maize silage, which was the highest in the present study, exceeded previously reported values [11]. Nevertheless, it was > 1 log_10_ cfu lower than the counts 7 days after exposure to air [11].

Concerning the tested bacteria species, *Escherichia coli* is commonly used as a hygienic indicator for fecal contamination in different matrices such as meat [12]. Lynn et al. [13] found *Escherichia coli* in 30% of cattle feeds, which are partly comparable to the tested enrichment materials in this study. However, in our study, *Escherichia coli* was not detected.

In a longitudinal study concerning Belgian dairy farms, 8.2% of unused sawdust bedding samples were contaminated with *K. pneumoniae* [14]. In contrast, in our study *Klebsiella* spp. was not detected in any of the tested materials. The absence of *Klebsiella* spp. and coliforms on the maize pellets contradicts a previous study [15] in which 2.8 log_10_ cfu/g of both microbes in unused pelleted corn cobs was detected.

Human yersiniosis is one of the most frequent foodborne infections in the EU, and pigs are an important reservoir for the main causative pathogen, *Yersinia* (*Y*.) *enterocolitica* [16]. Despite numerous studies on the subject, the infection source and farm environment impact, especially from living or inanimate vectors, remain unclear [16]. It is controversial whether the absence or sparse use of bedding material [17] or the presence of bedding material [18] is a risk factor for the prevalence of pathogenic *Y. enterocolitica* and antibodies to *Y. enterocolitica* in pigs. In our study, the analysis was not limited to *Y. enterocolitica* but was conducted with *Yersinia* spp. in general; still, there was no detection of *Y*. spp. in any enrichment material. This result is in accordance with Vilar et al. [17] who did not detect pathogenic *Y. enterocolitica* in straw, and a German study in which pathogenic *Y. enterocolitica* was not found in 830 environmental and feed samples [19].

*Salmonella* spp. is a common contaminant in feed raw materials, and therefore feed is one of the sources for introducing *Salmonella* into pig herds [20]. Control programmes for *Salmonella* have been implemented in several European countries and include financial penalties for herds with a high prevalence of *Salmonella* infection. Thus, it is important for pig farmers to minimise the introduction of *Salmonella* spp. onto farms. None of the tested enrichment materials contained *Salmonella* spp. in levels that could be detected by our methods even though a pre-enrichment step was included. Therefore, the tested materials do not represent a probable source of *Salmonella* infection.

With regard to antimicrobial resistance, MRSA is a major problem for human healthcare, and intensively reared pigs are one of the main reservoirs of livestock-associated MRSA [21]. Antibiotic resistant bacteria in addition to antibiotics and resistance genes can reach crop fields by land application of manure [22]. Furthermore, MRSA is regularly found in farm environments of positive herds and the air and soil outside the barns [23]. The risk of enrichment material contamination by these potential routes can be considered as low and did not lead to MRSA findings in the tested materials.

The only detected specific pathogens in the material tested in this study were *M. smegmatis*, *M*. *avium*, and *M*. *vulneris* in the hemp straw and peat. *M. smegmatis* belongs to the rapidly growing mycobacteria, which are saprophytes found in environments such as soil, water, and dust [24]. It is potentially pathogenic to humans and animals and can cause, for instance, cardiac-related infections, pulmonary diseases, or ulcerative skin lesions [24]. Due to the widespread occurrence of *M*. *smegmatis* in the environment and the rare cases of clinical disease, the presence of this microorganism in hemp straw should not be overestimated. *M. avium* and *M*. *vulneris* both belong to the *M*. *avium* complex [25]. *M. vulneris* can cause lymphadenitis and abscesses in immunocompetent patients [26]. *M. avium* is classified into four subspecies: (1) *avium*; (2) *hominissuis*; (3) *paratuberculosis*; and (4) *silvaticum*, each showing distinct host species and pathogenicity [25]. The detection of *M*. *avium* in unused peat is in accordance with previous studies, and peat is assumed to be a source of infection for pigs [26, 27]. In a Spanish study, *M*. *avium* was found in unused sawdust samples [28], which is neither supported by Agdestein et al. [27] nor by our present results. The most prevalent species in the mentioned studies is *M*. *avium* subsp. *hominissuis*, a microorganism that causes granulomatous lesions in pigs, leading to carcass condemnation at the abattoir and financial losses for farmers [28]. Matlova et al. [26] tested various decontamination methods for peat and demonstrated that steam treatment at 100 °C for 10 min effectively reduced mycobacterial contamination, though not completely eradicating the mycobacteria. The peat tested in our study had been heated at 65 to 70 °C for at least 10 min as certified by the producer. Obviously, this treatment is not sufficient to decontaminate peat, and therefore this commercial peat designated for piglets poses a risk for infecting animals with mycobacteria. It is debatable whether the infectious and granulomatous lesions should be interpreted as jeopardising the health in the legal sense as stipulated in the Council Directive 2008/120/EC [3]. Peat is a very heterogeneous material but is generally characterised by an acidic pH and humic substances [29]. The presence of microorganisms is crucial for the formation of peat; concurrently, some of them produce antibiotics [29]. All of these factors make peat a difficult environment for microbes. However, the aforementioned detection of mycobacteria is a major health risk, thus excluding the use of peat as a safe and hygienic enrichment material. As moulds are considered ubiquitous, the detected mould contamination is not remarkable. However, deterioration of organic material by moulds can minimise the palatability, which might result in reduced acceptance of the material, and most importantly, cause mycotoxin production [30]. Thus, further investigations on mycotoxins should be carried out. The hay from the farm had the highest mould count of all materials. Different hays can vary widely in their hygienic status, and a TVC of up to log_10_ 6.47 cfu/g and a mould count of up to log_10_ 6.67 have previously been reported [31]. The contamination in the present study is comparable to these studies, but to the authors’ knowledge, there is no evidence that this kind of contamination poses a health risk for pigs.

There are no microbiological thresholds set for enrichment material in pig husbandry to date. However, the thresholds for feeding material give good orientation even though the intake of enrichment material by the animals is assumed to be minor despite there being no exact data available on this [2]. Moreover, to standardize microbiological testing of enrichment material, proof of the sensitivity and the specificity of applied methods are important to avoid false negative results.

Analyses of other groups of potential pathogenic agents such as parasites or viruses were not part of this study. Nevertheless, transmission via the vector fomites and personnel of porcine reproductive and respiratory syndrome virus, one of the most important viruses in pig husbandry, has already been demonstrated [32]. Thus, introduction of other microorganisms through contaminated enrichment material might also be possible and scientific research in this field should be conducted.

## Conclusions

In conclusion, most of the tested organic materials did not harbor potentially pathogenic bacteria and were suitable as enrichment material for pigs. The hypothesis of the study, in which certain organic enrichment materials are a source of pathogenic bacteria that might cause infection in pigs, was confirmed for peat only. The *Mycobacteria* found in peat potentially jeopardises the health of pigs and might result in financial losses. Thus, using peat in pig farming is risky and not recommended by the authors. Whether all of the tested hygienic materials are appropriate enrichment materials from an ethological point of view and whether viruses pose a risk, requires further investigation.

## Methods

### Enrichment materials

A total of 21 different organic materials, described in Table 3, were examined in this study. Eighteen of them were commercially available in Germany. Wheat straw, hay, and maize silage were produced on the Farm for Education and Research in Ruthe of the University of Veterinary Medicine Hannover, Foundation. Four materials were made of wood, seven consisted of loose straw and hay, and another four tested products were made of compressed straw and hay. The remaining six materials varied widely in their nature and composition. The materials were purchased in February 2015, and samples for the laboratory analyses were subsequently taken. In June 2017, a second batch of peat was purchased to retry the test for mycobacteria since the samples had been overgrown by other bacteria in the first examination in 2015.

### Sample preparation

The water activity of all materials was measured with the Aquaspector AQS-31 (NAGY-Instruments GmbH, Gäufelden, Germany) at 20 °C. With the water activity (a_w_) calculated by the partial vapor pressure of water in a substance divided by the standard state partial vapor pressure of water, the availability of free water in the material is described. Values can range between 0 and 1. The DM content was determined by the Sartorius MA40 Moisture Analyzer (Sartorius AG, Göttingen, Germany). If needed, the materials were grinded. One gram of each material sample, pooled from samples taken from the outside and the inside of the –possibly grinded-material batch, was mixed with 50 mL phosphate-buffered saline (PBS) + 0.01% Tween® 20 (AppliChem GmbH, Darmstadt, Germany) and incubated for 30 min in a water bath at 25 °C while shaking at 140 min^− 1^. Subsequently, the suspension was vortexed by a Vortex-Genie® 2 (Scientific Industries, Inc., New York, USA) for 4 min at stage six. Serial dilutions were prepared with PBS + Tween from the supernatant fluid of the samples.

### Microbiological analyses

All microbiological tests, except for the analysis of mycobacteria were performed in technical triplicate. The grown colonies were counted to determine the titre of colony forming units per gramme DM of the sample (cfu/g DM). The limits of detection ranged between 2.68 (beet pulp) and 3.16 (maize silage) log_10_ cfu/g DM. For the analyses of TVC of aerobic mesophilic bacteria and moulds, 0.1 mL of prepared sample dilution was plated onto Blood Agar Base No.2 (Oxoid Deutschland GmbH, Wesel, Germany) and dichloran-glycerol (DG18) agar base (Oxoid) complemented with glycerol (Carl Roth GmbH + Co. KG, Karlsruhe, Germany) and 50 mg/L chloramphenicol (Carl Roth). Blood agar base was incubated aerobically for 48 h at 36 °C. The first colony count was performed after 24 h. DG18 Agar was incubated aerobically for 5–6 days at 25 °C. The first colony count was performed after 2 to 3 days. For the analyses of coliform count and *Escherichia coli,* aliquots of the prepared samples were plated on MacConkey Agar No.3 (Oxoid) and incubated aerobically for 48 h at 36 °C. The colonies of coliform bacteria and presumed *Escherichia coli* were counted. Subsequently, the presumed *Escherichia coli* were streaked on Columbia Agar plates with sheep blood (Oxoid) and incubated aerobically for 24 h at 36 °C. Quantification of *Klebsiella* spp. was also conducted via aerobic incubation on MacConkey Agar No.3 for 48 h at 36 °C. Presumed *Klebsiella* spp. colonies were streaked on Blood Agar Base No.2 and incubated aerobically for 48 h at 36 °C. Subsequently, the colonies were tested for oxidase and urease (Oxoid). *Yersinia* count was performed by plating the samples onto Cefsulodin-Irgasan-Novobiocin Agar (Oxoid) and incubating them aerobically for 48 h at 32 °C. Presumed *Yersinia* spp. colonies were streaked on Blood Agar Base No. 2 and incubated likewise. For the final confirmation of *E. coli*, *Klebsiella* spp. and *Yersinia* spp., an API® 20E test (bioMérieux Deutschland GmbH, Nürtingen, Germany) was carried out in accordance with the manufacturer’s instructions. The test results were analysed by the apiweb™-API 20E V5.0 software (bioMérieux SA, Marcy-l’Étoile, France). The samples were analysed for *Salmonella* spp. as stated in ISO 6579:2002/Amd.1:2007(E) [33]. For detecting MRSA, 25 g of enrichment material was mixed with 225 mL of Mueller-Hinton broth (Oxoid) with 6.5% NaCl (Carl Roth) and analysed as described by Schulz et al. (2012). The analysis of *Mycobacterium* spp. was conducted by the National Reference Centre for Mycobacteria (Borstel, Germany). One g of the material samples was mixed with 50 mL sterile phosphate buffer and agitated over night at ambient temperature. On the following day 10 mL of the supernatant was transferred to a centrifuge tube and a 5% solution of Tween® 80 was added to improve the concentration of mycobacteria in the subsequent centrifugation step. The further investigation was performed in accordance with Hillemann et al. [34]. Data analyses were performed descriptively with MS Excel 2010 (Microsoft Corporation, Redland, Washington, USA).
